# Editorial: Artificial intelligence in point of care diagnostics

**DOI:** 10.3389/fdgth.2023.1236178

**Published:** 2023-07-11

**Authors:** Massimo Martinelli, Davide Moroni, Ales Prochazka, Marija Strojnik

**Affiliations:** ^1^ Signals and Images Analysis Laboratory, Institute of Information Science and Technologies, National Research Council, Pisa, Italy; ^2^ Czech Institute of Informatics, Robotics and Cybernetics, Czech Technical University, Prague, Czechia; ^3^ Optical Research Center, León, Mexico

**Keywords:** artificial intelligence, point of care diagnostics (PoC), autonomous AI (AAI), computer-aided diagnostic method, automatic medical image detection, skin lesion segmentation network, microscopy

**Editorial on the Research Topic** Artificial intelligence in point of care diagnostics

Speeding up and improving the diagnosis process exactly where and when events occur is the goal of actual point of care (PoC) diagnostics. Besides progress in sensing technologies that pertain to multidisciplinary domains, including nanotechnologies, microfluidics, and advanced materials, it is envisaged that PoC diagnostics can significantly benefit from a tighter interplay with artificial intelligence (AI). The interdisciplinary impact of AI is closely related to the general area of digital signal processing, forming an integrating platform for different applications and unifying their background based on computational intelligence and machine learning (ML). This approach follows the ideas of Leibniz presented in history, trying to interconnect researchers of different narrow areas who lost their ability to communicate (1).

Indeed, AI and ML can lead to methods for integrating, analyzing, and understanding multimedia data from a plethora of different devices. In addition, multivariate methods can correlate the current patient status with the previous history, adapting the findings to their personal history, in line with a more personalized and adaptive approach to care and favoring a more accurate prediction of future status.

To this end, there is a need to explore different research directions in AI and PoC diagnostics. From one side, AI paradigms can be embedded into PoC testing devices, extending their capabilities and making possible analyses otherwise not viable, e.g. those including image analysis. This can lead to a convergence of pervasive computing and PoC diagnostics. Similarly, networks of local devices can be devised, taking advantage of distributed AI: wearable sensors and portable devices can communicate in an ecosystem, and their data can be cumulatively and coherently processed. Finally, AI can be decentralized, also considering a cloud-based approach, extending the capabilities of PoC diagnostics over the computational continuum. For instance, with a timely decentralized survey, PoC may allow the detection of anomalies that, once integrated with previously collected data and anamnesis, with the further purpose of a quality check to use reliable data, can be classified by AI methods. Immediately, the system can then alert the user and their caregivers. Moreover, specific assistance networks can guarantee control and rescue over the territory. Even if the cost of PoC devices is high, it reduces indirect costs and saves lives.

On the basis of such consideration, it has been our aim to collect in a Research Topic multidisciplinary contributions to *Artificial Intelligence in Point of Care Diagnostics* and, eventually, after a careful revision, five articles were included and published.

The computer-aided diagnostic method, including X-rays-based techniques, is one of the economical and safe options to diagnose disease, in particular pneumonia. A challenge to the currently existing models for the diagnosis of pneumonia has been feature extraction from the clinical pneumonia X-ray dataset. Four authors from China address this research problem by implementing techniques in AI: D. Yao and Z. Xu, from the State Key Laboratory of Reliability and Intelligence of Electrical Equipment at Hebei University of Technology, Tianjin, and Y. Lin and Y. Zhan, from the Department of Radiology, at the Hainan Women and Children’s Medical Center, Haikou. Yao et al. describe a two-step process in their article titled *Accurate and intelligent diagnosis of pediatric pneumonia using X-ray images and blood testing data*. They propose a two-stage training multimodal pneumonia classification method combining X-ray images and blood testing data, which improves the image feature extraction through a global-local attention module. They conclude that the two-stage strategy can reduce the misdiagnosis rate of the pneumonia model. They furthermore find that the data gap between bacterial pneumonia and viral pneumonia is very large when bacterial pneumonia and viral pneumonia are indistinguishable using their method.

Autonomous AI has the potential to reduce disparities, improve the quality of care, and reduce costs by improving access to specialty diagnoses at PoC. Diabetes and related complications incorporate a significant source of health disparities. Vision loss may be a complication of diabetes, supporting annual eye exams for prevention. Prior to the use of autonomous AI, store-and-forward imaging approaches diabetes-related eye exams were not frequent. The US Federal Food and Drug Administration recently approved an AI-based system to diagnose diabetic retinopathy (including macular edema) without a specialist physician overread at the point of care. Goldstein et al. working at Digital Diagnostics, Coralville, Iowa, United States wrote a comprehensive review to identify common workflow themes leading to the successful adoption of the AI-based system. They identify the determinants for scalable adoption of autonomous AI in the detection of diabetic eye disease in diverse practice types and key best practices learned through the collection of real-world data. They propose best practices upon the evaluation of four health centers, measured as the attainment number of exams per month using the autonomous AI system against targets set for each health center. They argue that attainable best practices can be generalized to other autonomous AI systems in front-line care settings, thereby increasing patient access, improving the quality of care, and addressing health disparities.

Automatic medical image detection utilizes AI techniques to accurately and efficiently detect lesions in medical images. It is a crucial task in computer-aided diagnosis (CAD) systems and can be integrated into portable imaging devices for intelligent PoC diagnostics. Feature Pyramid Networks (FPN) are commonly used deep-learning-based models for this purpose. However, FPN-based medical lesion detection models face two challenges: the object position offset problem and the degradation problem of IoU-based loss. To address these issues, in Xu et al. an international group of researchers from China and the UK, propose a novel FPN-based backbone model, i.e., Multi-Pathway Feature Pyramid Networks with Position Attention Guided Connections and Vertex Distance IoU (abbreviated as PAC-Net and VDIoU respectively), to replace vanilla FPN for more accurate lesion detection. They conduct extensive experiments on the Deeplesion dataset, a public medical image detection dataset. The results demonstrate that PAC-Net outperforms all existing FPN-based depth models in terms of lesion detection evaluation metrics. Furthermore, the proposed PAC module and VDIoU loss proved to be effective and essential for achieving superior performance in automatic medical image detection tasks. Additionally, the VDIoU loss exhibits faster convergence compared to existing IoU-based losses, making PAC-Net an accurate and highly efficient 3D medical image detection model.

In Bai and Zhou another kind of lesion, namely skin lesions, is addressed, focusing on automated segmentation of dermatoscopy images, a task that plays a vital role in early skin cancer diagnosis. The complexity and indistinct boundaries of skin lesions make this task challenging. In this study, R. Bai and M. Zhou, affiliated with the University of Chinese Academy of Sciences and the Department of Dermatology, China-Japan Union Hospital of Jilin University, Changchun, China, propose an innovative skin lesion segmentation network called SL-HarDNet. HarDNet serves as the backbone, enabling the network to learn more robust feature representations. Additionally, they introduce three powerful modules: the cascaded fusion module (CFM), the spatial channel attention module (SCAM), and the feature aggregation module (FAM). Briefly, the CFM combines features from different levels, effectively integrating semantic and location information of skin lesions. SCAM captures crucial spatial information, while FAM successfully fuses cross-level features. The high-level semantic position information features obtained from FAM are then reintegrated with CFM features to enhance the model’s segmentation performance. The authors evaluate and compare SL-HarDNet with state-of-the-art skin lesion segmentation methods on the challenge datasets ISIC-2016&PH2 and ISIC-2018. The experimental results consistently demonstrate that SL-HarDNet outperforms other segmentation methods, achieving the best performance in skin lesion segmentation.

Microscopy is another important domain in which AI can provide systems and tools to ease and make the diagnostic process more accurate. An example is reported in the article by Li et al. from the Sino-European School of Technology at Shanghai University and the School of Mechatronic Engineering and Automation at Shanghai University. They address the detection and analysis of circulating tumor cells (CTCs), which are crucial for precise cancer diagnosis and prognosis assessment. Traditional methods that rely on isolating CTCs based on physical or biological features are labor-intensive and unsuitable for rapid detection. Additionally, current intelligent methods lack interpretability, leading to diagnostic uncertainty. To address these challenges, the authors propose an automated method that utilizes high-resolution bright-field microscopic images to gain insights into cell patterns. Their method achieves precise identification of CTCs by employing an optimized single-shot multi-box detector (SSD)-based neural network with an integrated attention mechanism and feature fusion modules. Compared to conventional SSD systems, their method exhibits superior detection performance with a recall rate of 92.2% and a maximum average precision (AP) value of 97.9%. Notably, they combine the optimal SSD-based neural network with advanced visualization technologies, namely, gradient-weighted class activation mapping (Grad-CAM) for model interpretation and t-distributed stochastic neighbor embedding (T-SNE) for data visualization. The work thus demonstrates the outstanding performance of the SSD-based neural network for identifying CTCs in the human peripheral blood environment, offering great potential for early cancer detection and continuous monitoring of cancer progression.
